# Anti-Virulence Strategy against the Honey Bee Pathogenic Bacterium *Paenibacillus larvae* via Small Molecule Inhibitors of the Bacterial Toxin Plx2A

**DOI:** 10.3390/toxins13090607

**Published:** 2021-08-29

**Authors:** Julia Ebeling, Franziska Pieper, Josefine Göbel, Henriette Knispel, Michael McCarthy, Monica Goncalves, Madison Turner, Allan Rod Merrill, Elke Genersch

**Affiliations:** 1 Department of Molecular Microbiology and Bee Diseases, Institute for Bee Research, 16540 Hohen Neuendorf, Germany; julia.ebeling@hu-berlin.de (J.E.); franziska_pieper@gmx.de (F.P.); josefine.goebel@hu-berlin.de (J.G.); h.knispel@lm.mv-regierung.de (H.K.); 2 Department of Molecular and Cellular Biology, University of Guelph, Guelph, ON N1G 2W1, Canada; mmccar07@gmail.com (M.M.); mgonca06@uoguelph.ca (M.G.); mturne09@uoguelph.ca (M.T.); rmerrill@uoguelph.ca (A.R.M.); 3 Institute of Microbiology and Epizootics, Faculty of Veterinary Medicine, Freie Universität Berlin, 14163 Berlin, Germany

**Keywords:** ADP-ribosylation, small molecule inhibitor, anti-virulence strategy, bacterial toxin, virulence factor, *Paenibacillus larvae*, American Foulbrood

## Abstract

American Foulbrood, caused by *Paenibacillus larvae*, is the most devastating bacterial honey bee brood disease. Finding a treatment against American Foulbrood would be a huge breakthrough in the battle against the disease. Recently, small molecule inhibitors against virulence factors have been suggested as candidates for the development of anti-virulence strategies against bacterial infections. We therefore screened an in-house library of synthetic small molecules and a library of flavonoid natural products, identifying the synthetic compound M3 and two natural, plant-derived small molecules, Acacetin and Baicalein, as putative inhibitors of the recently identified *P. larvae* toxin Plx2A. All three inhibitors were potent in in vitro enzyme activity assays and two compounds were shown to protect insect cells against Plx2A intoxication. However, when tested in exposure bioassays with honey bee larvae, no effect on mortality could be observed for the synthetic or the plant-derived inhibitors, thus suggesting that the pathogenesis strategies of *P. larvae* are likely to be too complex to be disarmed in an anti-virulence strategy aimed at a single virulence factor. Our study also underscores the importance of not only testing substances in in vitro or cell culture assays, but also testing the compounds in *P. larvae*-infected honey bee larvae.

## 1. Introduction

The Western honey bee (*Apis mellifera*) is considered the most economically valuable pollinator and is responsible for the pollination of many fruits, vegetables and stimulant crops [1,2,3,4,5]. Maintaining honey bee health is therefore critical to human food security. This realization has led to increased scientific interest in honey bee health and honey bee diseases. The major threat to honey bee health is the ectoparasitic mite *Varroa destructor* [6,7] together with the viruses vectored by the mite, most importantly deformed wing virus (DWV) [8,9,10]. Other relevant honey bee diseases are nosemosis, a diarrhea in adult bees caused by the microsporidia *Nosema apis* and *N. ceranae* [11,12,13,14,15,16,17], and the bacterial brood diseases European Foulbrood (EFB) and American Foulbrood (AFB), caused by *Melissococcus plutonius* [18,19,20] and *Paenibacillus larvae* [21,22,23], respectively.

AFB, caused by the Gram-positive, spore-forming bacterium *P. larvae*, is the most destructive as well as a highly contagious brood disease which not only kills individual larvae but eventually the entire colony (for reviews see [23,24,25] and references therein). Hence, it is a notifiable honey bee epizootic in most countries and measures against this disease are regulated by law and most often include culling of overtly diseased colonies. The only infective form of *P. larvae* are the tenacious spores [26,27,28,29] which need to be orally ingested by very young (L1) larvae since larvae are only susceptible to *P. larvae* infection approximately 36 h after egg-hatching [30,31,32]. After ingestion, the spores germinate in the larval midgut. Vegetative *P. larvae* massively proliferate in the midgut lumen before they finally attack and breach the epithelial layer to enter the hemocoel, a step that coincides with larval death [33]. The larval cadavers then undergo complete degradation by *P. larvae* to the AFB-characteristic ropy mass which later dries out to a highly contagious scale containing the *P. larvae* spores that can again be transmitted to naive honey bee larvae via the nurse bees.

Different genotypes of *P. larvae* can be determined via repetitive element PCR (rep-PCR) with enterobacterial repetitive intergenic consensus (ERIC) primers [34] and are thereafter named ERIC genotypes [21]. All genotypes have been shown to cause AFB but differ in colony and spore morphology, metabolism, and virulence at both the individual larval and the colony level [21,35,36]. The *P. larvae* genotypes ERIC I and II are the most practically relevant genotypes as they are regularly isolated from current AFB outbreaks worldwide [37]. Comparative genome analysis of *P. larvae* ERIC I and ERIC II [38] revealed virulence factors common to both genotypes as well as virulence factors which differ between the genotypes and point to different pathogenesis strategies employed by *P. larvae* ERIC I and ERIC II (see recent reviews [23,39]). The key virulence factor for the species *P. larvae* is the chitin-degrading enzyme *Pl*CBP49, which belongs to the lytic polysaccharide monooxygenases (LPMOs) [40] and is used by *P. larvae* to degrade the peritrophic matrix lining and protecting the larval midgut epithelium [40,41]. For subsequent attack of the defenseless midgut epithelium, *P. larvae* ERIC I possesses two ADP-ribosylating AB toxins Plx1 and Plx2, which are genotype-specific virulence factors, and facilitate *P. larvae* ERIC I invasion into the hemocoel [42,43]. Plx1 is a phage-born, single-chain AB toxin. The N-terminal A-domain is homologous to the A-domains of the Pierisins, a group of ART toxins shown to act on DNA. Based on the similarity of the essential motifs for transferase activity between the Pierisins and Plx1, it was suggested although not yet experimentally proven that Plx1 acts on DNA [43]. Plx2 is a binary AB toxin with an enzymatically active A-domain showing properties of C3 exoenzymes. The ADP-ribosyl transferase activity of Plx2A targets RhoA in the host cell, thereby inhibiting cytokinesis, which can be deduced from the formation of bi-nucleated cells in an insect cell culture model [42]. These two toxins are not present in the genome of *P. larvae* ERIC II [38], which, on the other hand, exclusively expresses an S-layer protein (SplA) [44]. SplA mediates *P. larvae* adhesion to the midgut epithelial cells, presumably as the first step of *P. larvae* ERIC II in initiating bacterial attack on the epithelium and invasion into the larval hemocoel [45].

Although AFB is a global threat to honey bees and many research efforts have focused on this disease for decades, no cure has yet been found. Still, most authorities consider the burning of diseased colonies the only effective measure to prevent the further transmission and spread of the disease. In some countries, such as the USA and Canada, American Foulbrood is preventatively treated with antibiotics which oppress disease outbreaks but do not kill the bacteria because of *P. larvae*’s ability to form highly persistent spores. Unfortunately, the use of antibiotics is a double-edged sword as antibiotic-resistant strains of *P. larvae* have already been detected [46,47,48,49,50]. Moreover, restraining AFB outbreaks via the application of antibiotics to honey bee colonies is prohibited in many countries (e.g. member states of the European Union). For these reasons, a sustainable treatment against AFB is urgently needed.

So far, several approaches to find effective measures against AFB have been investigated, e.g., the application of essential oils [51], various plant extracts [52,53,54,55,56], synthetic indoles against spore germination [57], bacteriophage therapy [58,59,60,61,62], phage lysins [63], breeding for hygienic behavior [64], an integrated management strategy for the prevention of outbreaks [65], or supplementation with beneficial microbiota [66]. Unfortunately, however, none of these methods have yet shown to be fully effective in the field.

A promising alternative to the approaches listed above, and especially to antibiotic treatment, is the development of anti-virulence strategies to combat the currently untreatable *P. larvae* infection of honey bee brood. In contrast to conventional antibiotics which interfere in essential bacterial functions and either kill the bacteria (bacteriocidal) or inhibit growth (bacteriostatic), anti-virulence strategies target only pathogenicity- or virulence-associated mechanisms of the bacteria to be inhibited but leave survival- and fitness-related traits unaffected. In such an anti-virulence therapeutic approach, the pathogen only gets disarmed and not killed, and thus this strategy circumvents the direct selection pressure on the pathogen to produce resistant strains. Blocking bacterial virulence factors with small molecule inhibitors is therefore considered one possible strategy to combat bacterial infections as an alternative to antibiotics in the emerging field of antibiotic resistance (as reviewed by [67,68,69,70,71]). A further advantage of virulence factor inhibition is that the microbiome of the host does not get affected, in contrast to the use of antibiotics. Furthermore, targeting virulence factors also broadens the repertoire of bacterial targets by not only affecting essential factors for growth and survival of the pathogen (as reviewed by [67,68]). Strategies for the virulence inhibition currently under investigation are (i) the interference in the regulation of virulence factor expression, (ii) the interference in systems involved in virulence factor assembly, (iii) the interference in biofilm formation and bacterial adhesion, and (iv) a direct interference with the virulence factors such as the inhibition of bacterial toxin function or toxin delivery (see reviews by [67,68,69,70] and references therein). Compounds used for the inhibition of virulence factors already described include antibodies, nanoparticles, bioactive peptides, and small molecules, the latter being either synthetics screened from chemical libraries or natural compounds derived from plant extracts (as reviewed by [69]).

Targeting bacterial exotoxins in anti-virulence strategies is especially promising since toxin-producing pathogens heavily rely on these virulence factors for tissue damage, cellular malfunction, or destruction. Therefore, the deletion of toxin genes or the inhibition of toxin function normally results in avirulence or markedly reduced virulence. Virulence of *P. larvae* ERIC I has been shown to depend on the activity of two AB toxins, Plx1 and Plx2 [43]. Plx2 is a binary AB-toxin with a C3-like enzymatically active A-subunit, Plx2A. Plx2A has been verified as a virulence factor in exposure bioassays with honey bee larvae [43] and has been shown to ADP-ribosylate RhoA and cause bi-nucleated cells and vacuolization in an insect cell culture model [42]. Several inhibitors that target ADP-ribosyltransferase activity have been successfully identified [72,73,74,75,76]. However, to our knowledge, the only example so far for an inhibitor targeting the in vitro enzymatic activity of C3-like toxins is the small molecule inhibitor M3 [77], a substituted pyrazolo-pyrimidine backbone linked to a piperidine ring with a methanesulfonamide substituent. This inhibitor was identified from a library of small molecule toxin inhibitors that was previously generated from a virtual screen against a high-resolution structure of iota toxin in complex with NADH, as a representative bART toxin [76]. The inhibitor M3 was shown to be effective against Vis toxin from *Vibrio splendidus* [76] as well as against C3bot1 from *Clostridium botulinum* and C3larvin toxin from *P. larvae* [77].

In the present study, we investigated Plx2A as a target for anti-virulence therapy of *P. larvae* ERIC I infections. The Plx2A glycohydrolase (GH) activity [42], i.e., a side reaction of ADP-ribosyltransferases with water as an acceptor of the ADP-ribose moiety in the absence of the RhoA substrate, was exploited as measure for enzymatic activity. Herein, we report on the testing of the synthetic small molecule inhibitor M3 [77] and two natural inhibitors derived from plant extracts, Acacetin and Baicalein, as putative candidates for Plx2A toxin inhibition. Acacetin is a 4′-O-methylated flavone that is found in a variety of plants including *Robinia pseudoacacia* (black locust tree). It has been shown to exhibit inhibitory effects against several enzymes including glutathione reductase, aldose reductase, acetylcholinesterase, cyclo-oxygenase and xanthine oxidase enzymes. Structure-activity relationship studies showed that the hydroxyl groups at C-5/C-7, the double bond at C-2/3 and the methoxy group at the C-4′ are the key components for Acacetin biological activity [78]. Baicalein is a trihydroxy flavone isolated from *Sutellaria baicalensis* (Chinese skull cap) which is a flowering plant in the Lamaicea family (mint and sage) and it has been used extensively in Chinese medicine. Baicalein is an inhibitor of certain types of lipoxygenases and also has anti-inflammatory effects. Furthermore, it inhibits the CYP2C9 enzyme in the cytochrome P450 system that metabolizes drugs in humans [79]. We took a three-tiered approach and first tested the inhibitory compounds in enzyme assays for their ability to inhibit Plx2A GH activity and determined their IC_50_ values. We then analyzed the in vivo inhibitory activity of the small molecules against Plx2A in an insect cell culture model. Finally, we tested the efficacy of the inhibitors in experimentally *P. larvae*-infected honey bee larvae. All three small molecules inhibited Plx2A enzyme activity and M3 and Acacetin protected cultured cells against Plx2A intoxication, but neither the synthetic nor the plant-derived toxin inhibitors showed any effect on larval mortality in *P. larvae* ERIC I-infected larvae. This result suggests that the pathogenesis strategies of *P. larvae* are likely too complex to be abrogated in a simple anti-virulence strategy aimed at a single virulence factor. Furthermore, our results underpin the importance of not only testing putative therapeuticals in in vitro or cell culture-based assays, but also testing the compounds in the system that exhibits the *bona fide* disease state, that is, in *P. larvae*-infected honey bee larvae.

## 2. Results

### 2.1. Inhibition of Plx2A GH Activity by Synthetic and Plant-Derived Small Molecules

**Figure 1:**
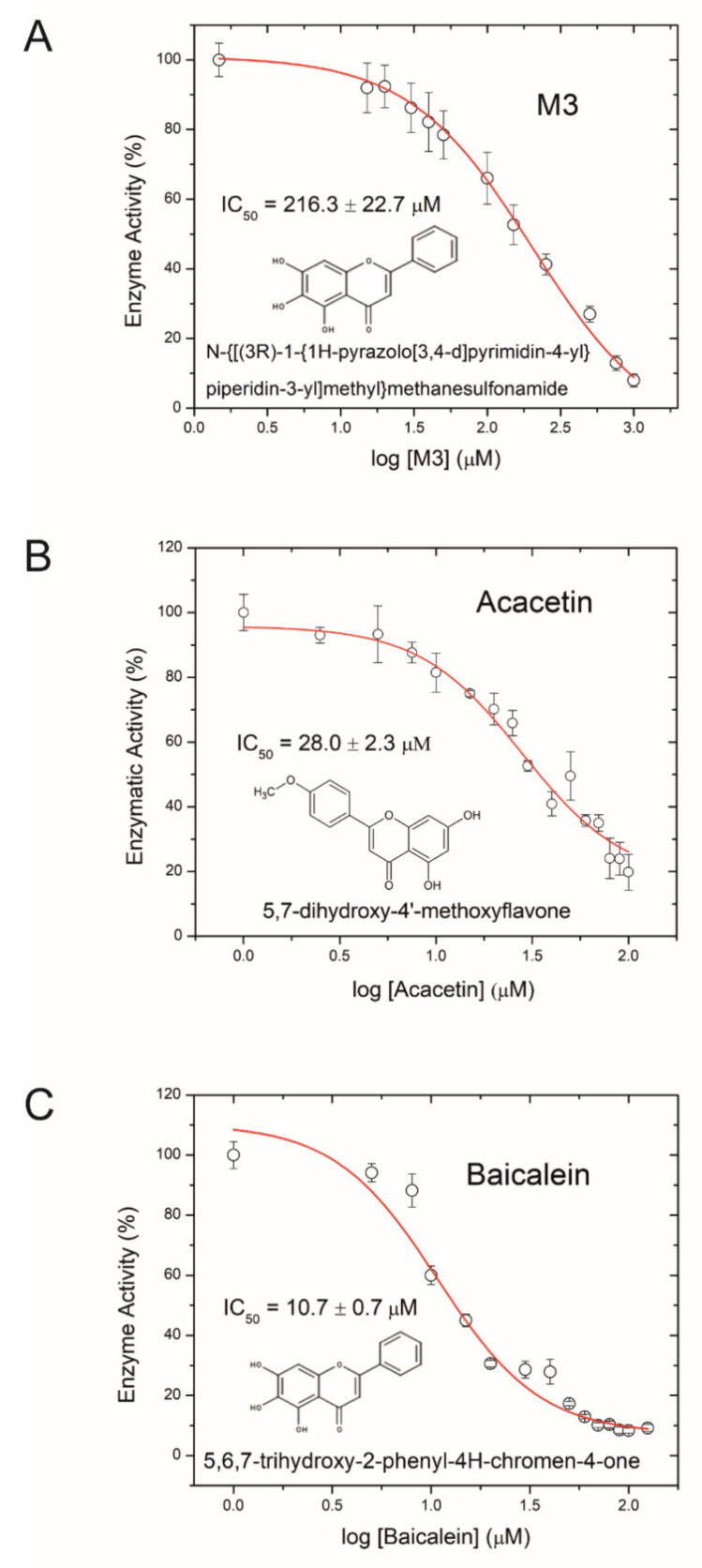
Inhibition of Plx2A GH-activity. (**A**) Dose-response curve for the synthetic inhibitor M3, (**B**) dose-response curve for the plant-derived inhibitor Acacetin, and (**C**) dose-response curve for the plant-derived inhibitor Baicalein on Plx2A GH activity. In the presence of increasing concentrations of inhibitor, Plx2A loses its GH activity indicative of an inhibition of enzyme function. Symbols indicate the mean of three experiments with three measurements each ± SEM.

Aiming to develop an anti-virulence therapeutic approach against AFB, we tested the small molecule inhibitor M3 for its ability to inhibit Plx2A activity. M3 was recently derived from a virtual screen of a synthetic small molecule library against iota toxin [76] and shown to be active against other C3-like toxins [77]. A dose-dependent reduction in Plx2A GH activity was used as a measure for successful toxin activity inhibition. The inhibitor concentration at which the enzymatic activity is reduced to 50% (IC_50_) was determined. The IC_50_ of M3 was 216.3 ± 22.7 µM (Figure 1A). This result identified M3 as being potent against Plx2A and suggested that M3 might be a useful lead compound for developing AFB anti-virulence strategies.

Next, we tested several flavonoids from a natural products library in the GH enzyme activity assay for inhibition of Plx2A. Two small molecules inhibiting GH activity were identified: Acacetin (5,7-dihydroxy-4′-methoxyflavone), an O-methylated flavone isolated from *Robinia pseudoacacia* (black locust tree), and Baicalein (5,6,7-trihydroxyflavone), a flavone isolated from *Scutellaria baicalensis* (Chinese skullcap), a flowering plant in the Lamaicea family. The IC_50_ values for Acacetin and Baicalein were 28.0 ± 2.3 µM, and 10.7 ± 0.7 µM, respectively (Figure 1B,C); hence, these two natural compounds were more potent inhibitors of Plx2A GH activity than the synthetic molecule M3. Importantly, it was determined that these flavonoids were reversible inhibitors of Plx2A activity that exhibit non-competitive inhibition, denoting that these compounds do not compete with the NAD^+^ substrate for the Plx2A toxin active site but rather bind to a distinct site on the enzyme (data not shown). However, the location and nature of this binding site has not yet been determined.

### 2.2. Inhibition of Plx2A Effect in an Insect Cell Culture Model

**Figure 2:**
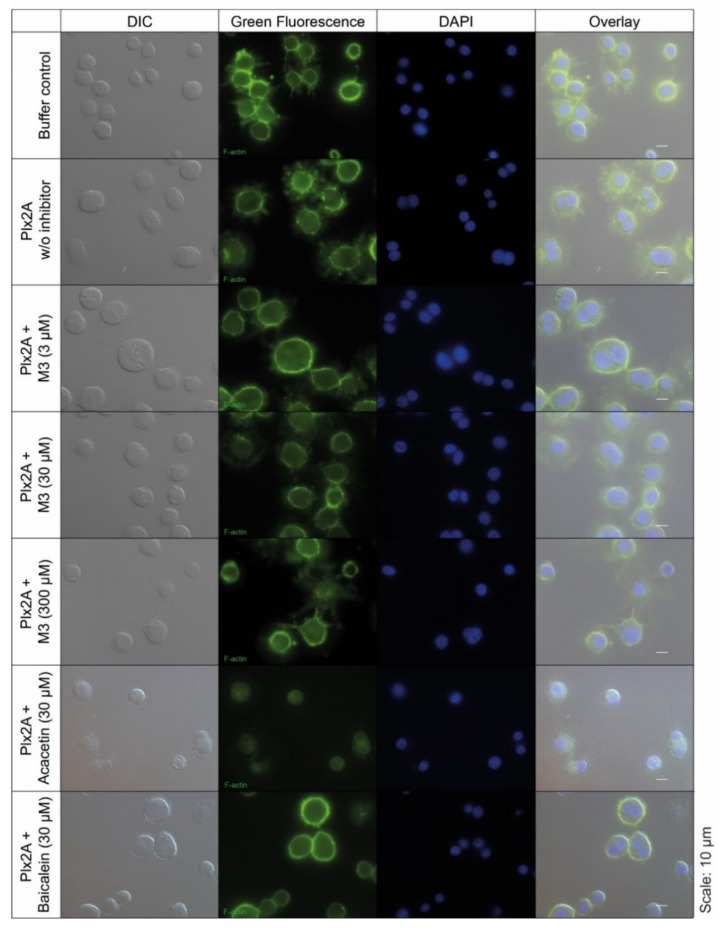
Tn5 insect cell culture assay with Plx2A and inhibitors. The cells were incubated with Plx2A and inhibitors for two days, and afterwards DyLight 488 phalloidin- and DAPI-stained to visualize the F-actin cytoskeleton and the cell nuclei, respectively. Bi-nucleated cells are indicative of Plx2A activity. Mono-nucleated cells comparable to the controls are indicative of inhibitor activity as was the case for M3 at 300 µM and Acacetin at 30 µM.

**Figure 3:**
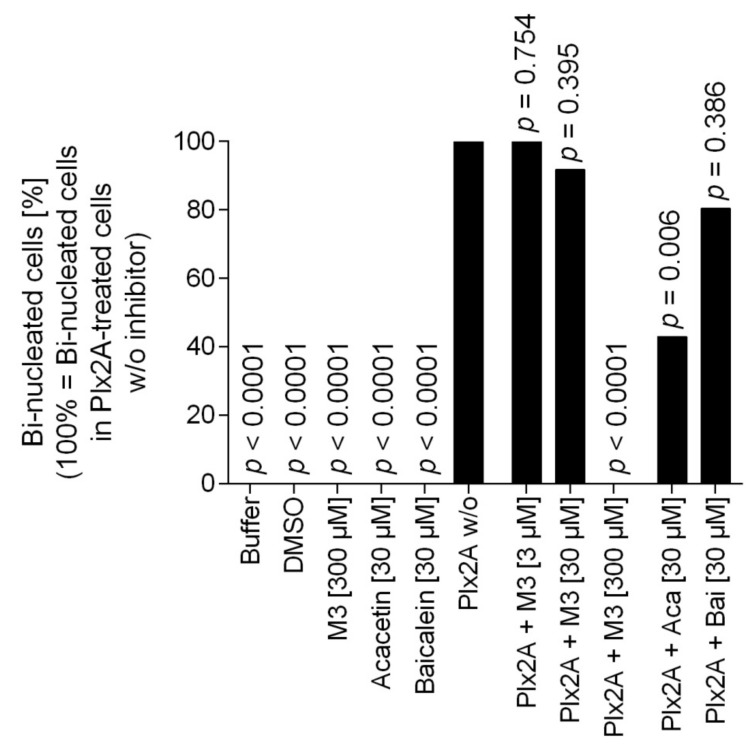
Quantification of bi-nucleated cells in the Tn5 insect cell culture assay. Per treatment group, around 100 cells were analyzed for the number of nuclei. The percentage of bi-nucleated cells in the treatment group that received only Plx2A without any inhibitors was set to 100% and the reduction in the groups that received Plx2A with inhibitors was then determined. Bars depict the percentage of bi-nucleated cells relative to the treatment with Plx2A alone without any inhibitor. The data was analyzed with pairwise chi-square tests with Bonferroni correction comparing all treatment groups to the treatment with Plx2A without inhibitor to check for a reduction in the number of bi-nucleated cells.

Next, we tested the efficacy of the three toxin inhibitors in an insect cell culture model. Currently, no suitable honey bee cell line is available, but in a recent study the insect cell line Tn5 derived from the cabbage looper *Trichoplusia ni* (Lepidoptera) was established as a suitable cell culture model for Plx2A activity [42]. To test the ability of the identified compounds to inhibit Plx2A toxin activity in cultured cells, Tn5 cells were incubated together with Plx2A and the different inhibitors for a time of 48 h. The cells were fixed and stained with DAPI and Phalloidin-DyLight 488 to enable the visualization of the cell nuclei and the actin cytoskeleton, respectively (Figure 2). As expected, treating the cells with Plx2A induced bi-nucleated cells, visible in fluorescence microscopy (Figure 2) and quantified by counting (Figure 3), confirming the already described interference of Plx2A with cytokinesis in Tn5 cells [42] and substantiating the usefulness of Plx2A-treated Tn5 cells as model and positive control. The synthetic inhibitor M3 was tested on Plx2A-treated cells at three different concentrations. A significant effect on the occurrence of Plx2A-induced bi-nucleated cells was observed at a final concentration of 300 µM (Chi-square test with Bonferroni correction, *p*_(M3, 300 µM)_ < 0.0001): Only mono-nucleated Tn5-cells were present (Figure 3) and cell morphology was normal (Figure 2), indicating that the M3 inhibitor protected the cells from Plx2A intoxication. Lower concentrations did not show a significant inhibitory effect on toxin activity (Figure 3) (Chi-square test with Bonferroni correction, *p*_(M3, 30 µM)_ = 0.395; *p*_(M3, 3 µM)_ = 0.754).

Testing the plant-derived inhibitors on Plx2A-treated cells revealed that Acacetin caused a significant reduction in bi-nucleated cells at a final concentration of 30 µM (Chi-square test with Bonferroni correction, *p* = 0.006), whereas no significant reduction was observed when Baicalein was added at a final concentration of 30 µM (Chi-square test with Bonferroni correction, *p* = 0.386). Hence, Acacetin can protect Tn5 cells from intoxication by Plx2A, but Baicalein is not capable of this protection activity. Testing final concentrations of 300 µM was not possible for Acacetin and Baicalein, because these concentrations were cytotoxic (data not shown).

### 2.3. Testing the Synthetic Small Molecule Inhibitor M3 and the Plant-Derived Small Molecules Acacetin and Baicalein in Honey Bee Larvae Experimentally Infected with P. larvae

**Figure 4:**
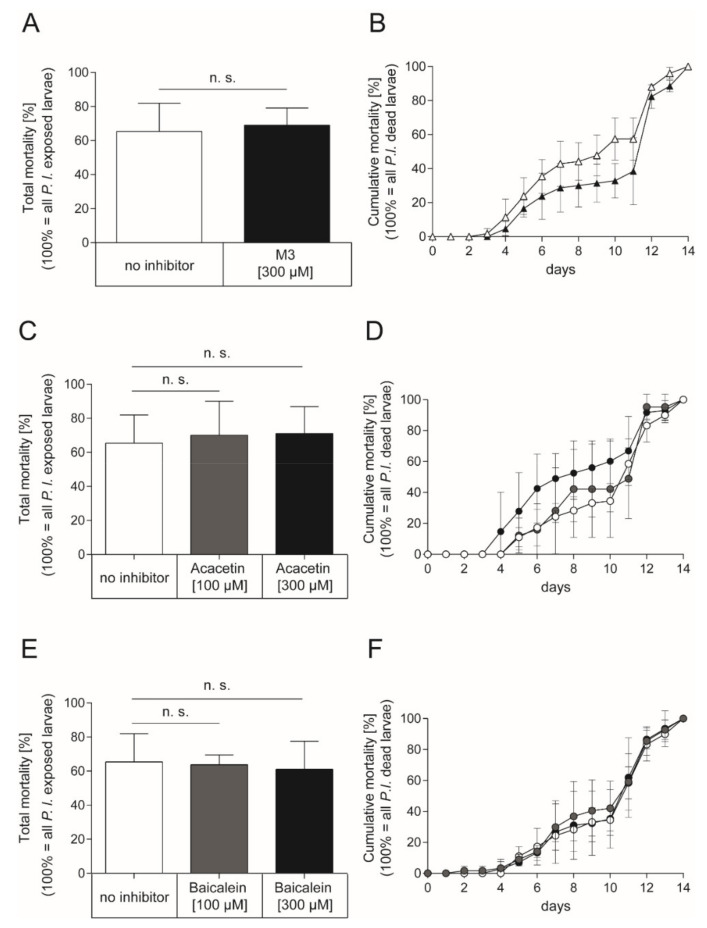
Exposure bioassay with honey bee larvae infected with *P. larvae* ERIC I strain ATCC 9545 and feeding with the synthetic inhibitor M3 or the plant-derived inhibitors Acacetin or Baicalein. (**A**) Total mortality and (**B**) cumulative mortality of honey bee larvae infected with *P. larvae* ERIC I (ATCC 9545) and fed with larval diet containing either no inhibitor (white) or the synthetic inhibitor M3 inhibitor at a final concentration of 300 µM (black). There was no significant difference between treatment and control in total mortality (unpaired *t*-test, *p* = 0.76) and cumulative mortality (two-way ANOVA, *p* = 0.14) (**C**) Total mortality and (**D**) cumulative mortality of honey bee larvae infected with *P. larvae* ERIC I (ATCC 9545) and fed with larval diet containing either no inhibitor (white) or the plant-derived small molecule inhibitor Acacetin at 100 µM (grey) or 300 µM (black). There was no significant difference between treatment and no inhibitor control in total mortality (unpaired t-test, Acacetin (100 µM): *p* = 0.77; Acacetin (300 µM): *p* = 0.69)) and cumulative mortality (2-way ANOVA, Acacetin (100 µM): *p* = 0.709, Acacetin (300 µM): *p* = 0.120). (**E**) Total mortality and (**F**) cumulative mortality of honey bee larvae infected with *P. larvae* ERIC I (ATCC 9545) and fed with larval diet containing either no inhibitor (white) or the plant-derived small molecule inhibitor Baicalein at 100 µM (grey) or 300 µM (black). There was no significant difference between Baicalein treated larvae and larvae that received no inhibitor in total mortality (unpaired t-test, Baicalein (100 µM): *p* = 0.88: Baicalein (300 µM): *p* = 0.76) and cumulative mortality (two-way ANOVA, Baicalein (100 µM); *p* = 0.575; Baicalein (300 µM); *p* = 0.887). The experiments were repeated three times (*n* = 3) with treatment groups of 30 larvae each, except for the experiment with infected larvae which received Acacetin at a concentration of 100 µM which was conducted with three times 10 larvae. The mean ± SD is shown; n.s., not significant.

As inhibition of Plx2A toxicity was successful for M3 and Acacetin in the insect cell culture model and a positive although not statistically significant trend was also observed for Baicalein, we tested the inhibitors in vivo in experimentally *P. larvae*-infected larvae. To this end, honey bee larvae were infected with *P. larvae* ERIC I (ATCC 9545) at a concentration of 150 cfu/mL resulting in 60–70% AFB-mortality (LC_60–70_) as described in the Materials and Methods section. To test the effect of the small molecule inhibitors on the infected honey bee larvae, the larval food was substituted with either M3 inhibitor or the plant-derived inhibitors for the whole feeding period of the experiment. The non-infected control groups received normal larval food or larval food with the corresponding concentration of DMSO. In the non-infected control groups, no honey bee larvae died from AFB. Corresponding to the concentrations used in the insect cell culture assay, the M3 inhibitor was applied at a final concentration of 300 µM in larval food. However, there was no significant effect on total mortality of infected larvae with M3-feeding compared to the total mortality of infected larvae which received no inhibitor (Figure 4A; unpaired *t*-test, *p* = 0.76) or cumulative mortality (Figure 4B; 2-way ANOVA, *p* = 0.14) of *P. larvae* ERIC I infected honey bee larvae. The plant-derived inhibitors, Acacetin and Baicalein, were also applied at a final concentration of 300 µM; corresponding to the final concentration of M3. There was also no significant effect on total mortality of the infected larvae which received Acacetin or Baicalein compared to the infected larvae that did not receive an inhibitor (Figure 4C; unpaired *t*-test, Acacetin: *p* = 0.69, Baicalein: *p* = 0.76) or cumulative mortality (Figure 4D; 2-way ANOVA, Acacetin: *p* = 0.12, Baicalein: *p* = 0.89) for both inhibitors. Furthermore, lower concentrations of Acacetin and Baicalein at 100 µM did not have a significant effect on the total mortality (Figure 4C; unpaired t-test, Acacetin: *p* = 0.77, Baicalein: *p* = 0.88) or cumulative mortality (Figure 4D; two-way ANOVA, Acacetin: *p* = 0.179, Baicalein: *p* = 0.575) compared to the mortality of infected larvae which received no inhibitor.

## 3. Discussion

AFB is a rather common yet serious and destructive bacterial disease of honey bees (for recent reviews see [23,24] and references therein). Currently, sustainable treatment strategies and effective drugs able to cure diseased colonies are lacking and the efficacy of antibiotics against spore-forming bacteria such as *P. larvae* is restricted because antibiotics are only active against vegetative bacteria.

An extremely promising alternative approach to antibiotic therapy is the development of anti-virulence strategies [80]. These strategies target pathogenesis or virulence-related bacterial traits and simply disarm the pathogenic bacteria instead of interfering with their survival or fitness. Among the most obvious bacterial virulence factors to be targeted by anti-virulence strategies are the exotoxins used by many pathogenic bacteria for manipulating or killing host cells. The virulence of *P. larvae* ERIC I has recently been shown to rely on the activity of two AB toxins, Plx1 and Plx2 [43]. The enzymatically active subunit Plx2A has been characterized in detail and was shown to be a C3-like toxin with ADP-ribosyltransferase activity [42]. In our attempt to develop an anti-virulence strategy for *P. larvae*, we therefore aimed to find inhibitors for Plx2A. We identified one synthetic (M3) and two plant-derived (Acacetin, Baicalein) compounds which inhibited the activity of Plx2A in an enzyme activity assay.

The synthetic inhibitor M3 has recently been identified as the first general inhibitor for the C3 subgroup of mono-ADP-ribosylating toxins [77]. M3 has also been shown to inhibit C3larvinA, a C3-like, ADP-ribosylating toxin expressed by a single orphan strain of *P. larvae* [77,81,82]. Considering the similarity between C3larvinA and Plx2A and the general ability of M3 to inhibit C3-like toxins, it was not surprising that it also showed inhibitory activity against Plx2A.

Plant extracts have been used by mankind for treating diseases since ancient times (as a recent review [83]) and the antimicrobial activity of plant juices and plant extracts has been scientifically investigated for decades [6,84,85]. However, the potential of plant-derived antimicrobial compounds as inhibitors of bacterial toxins and as agents in anti-virulence therapy, has only recently come into focus. They are now envisioned as a group of promising small molecule toxin inhibitory compounds. The two plant-derived flavonoids—Acacetin isolated from *Robinia pseudoacacia* (black locust tree) and Baicalein, isolated from *Scutellaria baicalensis* (Chinese skullcap)—which were identified as inhibitors of Plx2A in the GH enzyme activity assay, were even more potent inhibitors of Plx2A GH activity than the synthetic molecule M3.

However, when we tested these three promising small molecule Plx2A inhibitors in vivo in insect cell culture assays, only two of them (M3 and Acacetin) were able to protect the cells from Plx2A intoxication. Protection against Plx2A activity by M3 was achieved with 300 µM, whereas 30 µM Acacetin were sufficient to generate the same protective effect, that is to prevent formation of bi-nucleated cells as a result of Plx2A intoxication [42]. This was consistent with the higher potency of Acacetin compared to M3 in the GH enzyme activity assays. In these assays, Baicalein was even more potent than Acacetin and therefore, it was surprising that it did not inhibit Plx2A activity in insect cells. Unfortunately, higher concentrations could not be tested; it is assumed, however, that this would not change the results considering that Acacetin was less potent than Baicalein in the GH enzyme activity assays but was active in cell culture at 30 µM.

Since we could not explain the differences in the Plx2A inhibitory activities of the three small molecules, we tested the anti-virulence capacity of all three compounds in infected larvae, but none of the three compounds tested had a significant effect on the total and cumulative mortality of honey bee larvae infected with *P. larvae* ERIC I. That was rather surprising because it was previously shown that infecting larvae with a *P. larvae* gene inactivation mutant for the toxin subunit Plx2A resulted in a significant reduction albeit no complete loss in total larval mortality in exposure bioassays [43]. Therefore, we had expected that feeding the inhibitors would at least result in reduced mortality of *P. larvae* ERIC I infected larvae, similarly to the knockout effect. Additionally, a delay in the course of infection was considered possible as it was observed when larvae were infected with the gene inactivation mutant of the nonribosomal peptide synthetase/polyketide synthase (NRPS/PKS) cluster responsible for the production of the secondary metabolite paenilamicin (Pam) [86].

One possible explanation for the failure of these inhibitory compounds to protect the larvae from the progression of disease is that Plx2A is not the key or sole virulence factor of this genotype but only one of several virulence factors. Gene inactivation of Plx2A did not result in an avirulent strain of *P. larvae* ERIC I, but in a mutant strain with a 65% reduction in lethality [43]. Hence, feeding Plx2A inhibitors through an anti-virulence therapy was not expected to totally inhibit *P. larvae* virulence. That not even a slight inhibitory effect on mortality was observed when feeding M3, Acacetin or Baicalein could therefore be due to the complex set of virulence factors used by *P. larvae* ERIC I during pathogenesis. So far, Plx1, Plx2, *Pl*CBP49, and Pam have been experimentally proven in exposure bioassays as important virulence factors of ERIC I [40,43,86] and even more putative virulence factors were identified through whole-genome sequencing [38]. Therefore, a more promising anti-virulence strategy for combatting *P. larvae* infections might be the inhibition of a combination of virulence factors or the targeting of a single, key virulence factor. A promising candidate for the latter approach is the chitin-degrading enzyme *Pl*CBP49. Gene inactivation of *Pl*CBP49 in *P. larvae* ERIC I and ERIC II resulted in avirulent strains [40] and, hence, inhibiting *Pl*CBP49 through small molecules as part of an anti-virulence strategy should have a profound effect on pathogenesis. Moreover, such a strategy would be effective against both biologically relevant genotypes, *P. larvae* ERIC I and ERIC II, because *Pl*CBP49 is the key virulence factor of the species *P. larvae* [40].

Another possible explanation for the lack of inhibitory activity of M3, Acacetin and Baicalein in *P. larvae*-infected larvae are the conditions in the larval gut which are more complex than any cell culture model. In future studies, we will address the stability of the three compounds in the larval gut and analyze whether the pH or the digestive enzymes of the larva or even the enzymes secreted by *P. larvae* influence the compounds’ stability and activity. Depending on the results, the identified small molecules can serve as lead structures and based on the structure of the identified inhibitors, rational drug design approaches will enable the development of more stable and potent inhibitors formulated to allow their application in honey bee hives for the treatment of AFB.

As a more general conclusion, the results presented here emphasize the importance of conducting multi-stage studies when attempting to identify drug candidates for AFB treatment. Compounds that are active against virulence factors in in vitro enzyme assays or cell culture assays, as well as compounds that inhibit *P. larvae* in bacterial plate inhibition assays, are not necessarily active in infected larvae. We therefore claim that studies aiming to find a treatment against AFB not only include in vitro assays such as testing of the antimicrobial activity or inhibitory effect of a substance but also include testing the utility of the suggested treatment in vivo; at least in exposure bioassays with *P. larvae* infected honey bee larvae if not in experimentally infected mini colonies under appropriate precautionary and safety measures [36].

## 4. Materials and Methods

### 4.1. Recombinant Plx2A and Small Molecule Inhibitors

Plx2A was expressed as recombinant protein Plx2A-His_6_ in *E.coli* and purified by immobilized metal-affinity-chromatography (IMAC) and size-exclusion chromatography as described [42]. For further assays, the His_6_-tag was not removed and the recombinant protein Plx2A-His_6_ used in this study is simply referred to as Plx2A [42]. The synthetic small molecule inhibitor, M3, was commercially obtained from Molport (Riga, Latvia). The plant-derived inhibitors, Acacetin and Baicalein, were commercially obtained from TCI Deutschland GmbH (Eschborn, Germany).

### 4.2. Glycohydrolase (GH) Activity, Inhibitor Testing and Generation of IC_50_ Data

The GH activity of Plx2A was determined as previously described [42]. In brief, the GH activity assay is based on a 10-fold increase in fluorescence caused by the cleavage between nicotinamide and etheno (ε)-ADP-ribose of the fluorescent NAD^+^ analog ε-NAD^+^ [87,88]. For the M3 inhibitor, the fluorescence was measured in a FLUOstar Omega plate reader (BMG Labtech, Cary, NC, USA) with 300 nm excitation and 405 nm emission wavelength, and band passes of 5 nm. For M3 inhibitor testing, the concentrations of Plx2A and ε-NAD^+^ were kept stable at concentrations of 2.5 µM and 480 µM, respectively, in GH reaction buffer (20 mM Tris, 50 mM NaCl, pH 7.9). The final reaction volume was 100 µL in Corning^®^ 96 well clear flat bottom UV-transparent microplates (# 3635, Corning, NY, USA) sealed with clear tape. M3 was identified from an in-house library of inhibitors (M-series) that was generated from a virtual screen against a high-resolution structure of iota toxin in complex with NADH, as a representative bART toxin [76]. For M3 the inhibitor, concentration at which the enzymatic activity was reduced to 50% (IC_50_) was determined by titration with inhibitor concentrations between 0 µM and 1000 µM.

For the plant-derived flavonoid inhibitors, Acacetin and Baicalein, GH assays were conducted with a Cary Eclipse fluorescence spectrophotometer (Agilent Tech., Mississauga, Canada) with excitation wavelength of 305 nm and the emission wavelength of 405 nm and band passes of 5 nm. ε-NAD^+^ substrate was held at 400 μM and was mixed with 5 μM Plx2A in GH reaction buffer in a final reaction volume of 75 μL. Acacetin and Baicalein were dissolved in 100% DMSO and the final concentrations of Acacetin and Baicalein inhibitors in the reaction mixture ranged from 0 to 100 μM. Triplicate kinetic traces were collected for 10 min intervals and the reaction of the initial slope was recorded for each concentration of flavonoid inhibitor. An εAMP standard curve was obtained to convert fluorescent values to [εADP-ribose] formed per min. An IC_50_ dose-response curve was plotted and fitted with OriginPro-8 software using a dose-response function (OriginLab Corp, Northampton, MA, USA).

### 4.3. Insect Cell Culture and Actin and DAPI Staining

The insect cell culture assay was essentially performed as previously described [42]. The cell line BTI-Tn5B1-4 (Tn5) derived from the cabbage looper *Trichoplusia ni* (Lepidotera) was cultured at 27 °C in SF 900 II medium (Lonza, Basel, Switzerland) until confluency. Cells were counted with a Neubauer improved counting chamber and afterwards diluted to a concentration of 0.5 × 10^5^ cells/mL in medium supplemented with penicillin (250 IU/mL)- streptomycin (250 µg/mL) (Carl Roth GmbH & Co. KG, Karlsruhe, Germany). The cells were premixed with purified, sterile-filtered Plx2A in a final concentration of at least 1.0 µM with NaCl-Tris-buffer. These cells served as a positive control for toxin activity. For toxin inhibition, M3 inhibitor at final concentrations of 300 µM, 30 µM, or 3 µM, or the plant-derived inhibitors Acacetin or Baicalein both at a final concentration of 30 µM (a final concentration of 300 µM was cytotoxic; data not shown) were added to the pre-mix. As negative controls, cells were premixed with sterile filtered (i) NaCl-Tris-buffer or (ii) DMSO or (iii) M3 inhibitor (600 µM) alone. The premixed cell suspensions were applied to wells of sterile Cellstar 6- well tissue culture plates (Greiner Bio-One GmbH, Leipzig, Germany) which were each previously equipped with a sterile cover slip. The cells were incubated at 27 °C for two days. Actin staining was performed as described before [42]. Cells were fixed in Roti-Histofix 4% (Carl Roth GmbH & Co. KG) on the coverslip. The actin cytoskeleton of the cells was stained with Phalloidin Control, DyLight 488 conjugate (Invitrogen, Thermo Fisher Scientific, Darmstadt, Germany). The cell nuclei were stained with DAPI (4′,6-diamidino-2-phenylindole) as a component of the mounting medium ProLong Gold Antifade Mountant with DAPI (Invitrogen, Thermo Fisher Scientific). For microscopic analysis, a Nikon Ti-E Inverted Microscope (Düsseldorf, Germany) was used with the corresponding NIS Elements AR 3.10 Software (Laboratory Imaging, Nikon). Differential Interference Contrast (DIC) was used for light microscopy and a FITC (fluorescein isothiocyanate) or DAPI filter for the appropriate fluorescence detection.

For quantification of the mono- and bi-nucleated cells in the cell culture assays, about 100 cells per treatment were examined for the number of nuclei (either one or two nuclei). The data were analyzed with pairwise chi-square tests with Bonferroni correction comparing all of the treatment groups to the treatment with Plx2A without inhibitor to check for a reduction in the number of bi-nucleated cells in XLSTAT software, version 2019.3.2 (Data Analysis and Statistical Solution for Microsoft Excel, Addinsoft, Statcon, Witzenhausen, Germany).

### 4.4. Bacterial Strain and Culture Conditions

In this study, the *P. larvae* ERIC I strain ATCC 9545 was used. This strain has been thoroughly characterized before, including virulence analysis in exposure bioassays with honey bee larvae [21,33,35,43,89]. Bacteria cultivation was either performed on Mueller-Hinton-yeast-phosphate-glucose-pyruvate (MYPGP) or on Columbia sheep blood agar plates (CSA, Oxoid, Hampshire, UK) or in MYPGP liquid broth at 37 °C as described in the aforementioned papers. Generation of *P. larvae* spore suspensions and determination of spore concentrations via serial dilutions were essentially performed as previously described [35].

### 4.5. Exposure Bioassays with Inhibitor Feeding

Exposure bioassays were essentially performed as previously described [21,35,90]. First instar honey bee larvae were grafted into 24-well plates containing larval diet (3% (*w*/*v*) fructose, 3% (*w*/*v*) glucose, and 66% (*v*/*v*) royal jelly (Werner Seip Biozentrum GmbH & Co KG, Butzbach-Ebersgöns, Germany)). Honey bee larvae were exposed to *P. larvae* ATCC 9545 spore-spiked diet for the first 24 h; thereupon they returned to a normal larval diet. During the larval stages, the honey bee larvae were transferred daily to wells with fresh larval diet until the onset of pupation when they defecate and stop feeding. For pupal development, engorged larvae were transferred to wells lined with clean paper tissues and kept there until the end of the experiment. A lethal concentration (LC) of about 60% was achieved by applying a spore concentration of 150 cfu/mL. The naïve control group was fed with normal larval diet for the entire feeding period. The honey bee larvae were monitored daily for 14 days and transferred daily to wells with fresh larval diet until the start of pupation. Pupae were kept on clean filter tissues. Dead honey bees (larvae and pupae) were removed from the wells, streaked out on CSA plates, and incubated at 37 °C for two days to test for *P. larvae* infection. Only those larvae that died from *P. larvae*-infection were taken into account in the evaluation of larval mortality due to *P. larvae*. This experimental design allows for the monitoring of larval mortality due to both, *P. larvae* infection and manipulation or treatment (see below). Non-*P. larvae* induced larval mortality was below 15% in all assays. *P. larvae* was never isolated from uninfected control larvae. *P. larvae* monocultures grew only on plates from infected honey bee larvae. Thereof, DNA was extracted and *P. larvae* ERIC I was verified via PCR with Plx2A-specific primers c2-I_Sma F/c2-I_Not R [43]. Exposure bioassays were performed in three independent experiments with 30 larvae per treatment group each. The total mortalities were compared via unpaired *t*-test and the cumulative mortalities were analyzed via two-way ANOVA in GraphPad Prism 6.01.

To test the efficacy of the inhibitors as possible treatment for AFB, feeding with three different inhibitors (M3, Acacetin, Baicalein) was performed. To this end, honey bee larvae were kept either on normal larval diet, or on larval diet also containing M3 inhibitor with a final concentration of 300 µM corresponding to 0.12% (*v*/*v*) DMSO, or on larval diet containing either Acacetin or Baicalein with a final concentration of 100 µM and 300 µM corresponding to 0.02% (*v*/*v*) and 0.06% (*v*/*v*) DMSO each, respectively. The inhibitors were included in the larval diet throughout the whole feeding period of the experiment.

The control of the larval toxicity of the tested inhibitors is automatically part of the larval assays. The chosen spore concentration that caused around 60% of larval mortality due to *P. larvae* allows one to see both a decrease and an increase in larval mortality associated with applying the tested substances. No increase in non-*P. larvae*-mortality as well as no increase in *P. larvae*-induced mortality compared to the no-inhibitor control was observed in the groups treated with M3, Acacetin or Baicalein, ruling out larval toxicity of the tested substances and adverse side effects of the tested substances exclusively on infected larvae.

### 4.6. DMSO Effect on Vegetative P. larvae and P. larvae Spores

The inhibitors were dissolved in DMSO which is a polar, aprotic solvent commonly used to solve therapeutic and toxic agents that are insoluble or difficult to dissolve in water [91,92]. Furthermore, DMSO is commonly used as solvent for agents to be tested in cell culture models [93,94] and also in honey bee experiments [95,96,97,98,99] at low concentrations. However, higher concentrations of DMSO can have cytotoxic or other negative impacts on cultured cells [100,101,102] as well as a negative impact on honey bee development [97]; thus, the DMSO concentration should not exceed a specific threshold and the inclusion of appropriate DMSO controls is highly recommendable.

**Figure 5:**
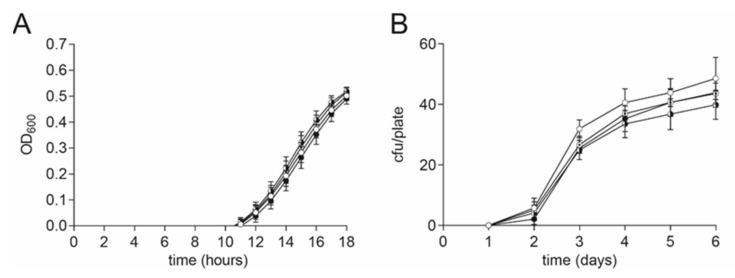
No DMSO effect on *P. larvae* vegetative cells and spores. (**A**) No significant difference in growth of vegetative *P. larvae* in MYPGP liquid culture was found under anaerobic conditions in the presence of different concentrations of DMSO (2-way ANOVA, *p* = 0.87). Symbols indicate the mean ± SEM from three biological replicates with three technical replicates each. (**B**) No significant influence on germination of *P. larvae* spores was detected on MYPGP agar plates containing different concentrations of DMSO (2-way ANOVA, *p* = 0.58). Symbols indicate the mean ± SEM from three biological replicates with two- three technical replicates each. (Circle colors: white: negative control; with dot: 0.004% (*v/v*) DMSO; black-white circle: 0.04% (*v/v*) DMSO; black circle: 0.4% (*v/v*) DMSO).

For this reason, we tested the effect of different DMSO concentrations on the growth of vegetative cells and also the germination of spores of the pathogen *P. larvae*. The growth of vegetative *P. larvae* in MYPGP liquid broth under the presence of different final concentrations of DMSO (0.4, 0.04, and 0.004% (*v*/*v*)) or no DMSO as a negative control was measured under anaerobic conditions. *P. larvae* was grown in a sterile flat-bottom 96-well-plate (Greiner Bio-One GmbH, Frickenhausen, Germany) in a Synergy HT plate reader (BioTek, Bad Friedrichshall, Germany) at 37 °C, shaking. The starting OD_600_ was 0.001. The OD_600_ was measured every hour for 18 h. Wells were covered with sterile filtered mineral oil for anaerobic conditions. The experiment was conducted with three biological replicates with three technical replicates each. DMSO did not have any significant effect on the growth of vegetative *P. larvae* (Figure 5A, 2-way ANOVA, *p* = 0.87).

A possible effect on the germination of *P. larvae* spores under the presence of DMSO was examined with MYPGP-agar plates containing different final concentrations of DMSO (0.4, 0.04, and 0.004% (*v/v*)) or no DMSO as a negative control. The *P. larvae* ATCC 9545 spore suspension was plated out after heat activation at 90 °C for 5 min. The plates were incubated upside down at 37 °C for 6 days. The colony forming units (cfu) per plate were counted every day. The experiment was conducted three times with two-three technical replicates per run. DMSO did not have a significant effect on the germination of *P. larvae* spores (Figure 5B, 2-way ANOVA, *p* = 0.58).

### 4.7. DMSO Effect on Honey Bee Larvae

**Figure 6:**
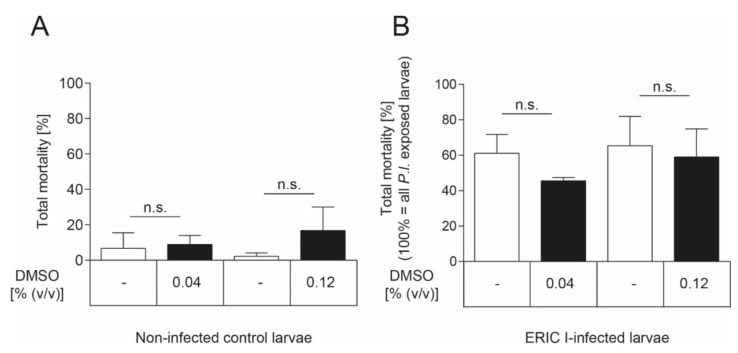
No DMSO effect on honey bee larvae. (**A**) There was no significant effect of DMSO on non-infected honey bee larvae in both concentrations tested (unpaired *t*-test, *p* > 0.05). None of the honey bee larvae neither was infected with *P. larvae* nor died from infection with *P. larvae*. (**B**) There was no significant effect of DMSO, in both concentrations tested, on the total mortality of honey bee larvae that died of *P. larvae* ERIC I-infection (unpaired *t*-test, *p* > 0.05). Only honey bee larvae that died from infection with *P. larvae* are included in this analysis. The experiments were repeated three times with 30 honey bee larvae per treatment group each. Bars with error bars represent the mean ± SD; n.s., not significant.

The inhibitor was dissolved in DMSO which showed a negative effect on honey bee brood development (more than 20% larval mortality) at concentrations above 0.2% (*v/v*) (data not shown). To demonstrate that the DMSO concentrations used in the experiments did not negatively affect larval survival, an uninfected and a *P. larvae*-infected control group were included in the exposure bioassay which both received larval diet supplemented with DMSO. These DMSO groups were compared to the uninfected groups and the infected larvae that received larval food without any additives, respectively. There was neither a significant effect of DMSO on the total mortality of non-infected (Figure 6A) nor ERIC I-infected (Figure 6B) honey bee larvae at both concentrations tested (unpaired *t*-test, *p* > 0.05) compared to the corresponding control group which received food without any additives.

## Data Availability

All data generated or analyzed during this study are included in this published article.
